# Piecewise Linear Strength Models for Analyzing Multiple Failure Mechanisms in Rocks Materials

**DOI:** 10.3390/ma17164102

**Published:** 2024-08-19

**Authors:** Shiqi Li, Yuan Li, Dongjue Fan, Liang Zhao, Litian Zhang

**Affiliations:** School of Civil and Resource Engineering, University of Science and Technology Beijing, Beijing 100083, China; m202221602@xs.ustb.edu.cn (S.L.); 18104835851@163.com (D.F.); m15833200311@163.com (L.Z.); zzyylltt@163.com (L.Z.)

**Keywords:** transformation of failure mode, rock material strength criterion, extensional-strain criterion, Paul-Mohr-Coulomb model

## Abstract

Rock materials failures are accompanied by the co-existence of various failure mechanisms, including rock fracturing, shearing, and compaction yield. These mechanisms manifest macroscopically as multiple failure modes and nonlinear strength characteristics related to stress levels. Considering the limitations of current rock mechanics strength theories, which are primarily derived from single failure mechanisms, this study evaluates the applicability of alternative strength theories. Based on the extensional-strain criterion and the PMC (Paul-Mohr-Coulomb) model, a piecewise linear strength model was proposed that is suitable for analyzing multiple failure mechanisms in rocks, revealing the intrinsic mechanisms of multi-mechanism rock material failure. A multiple failure mechanism strength model in the form of inequalities was proposed, using the generalized shear stress, mean stress, and stress Lode angle as parameters. Strength tests conducted on sandstone and granite rock material samples under different stress conditions revealed distinct piecewise linear strength characteristics for both rock types, validating the rationality and applicability of the multiple failure mechanism model. The findings construct a multi-mechanism failure model for rocks, providing enhanced predictive capabilities and aiding in the prevention of rock structural failures.

## 1. Introduction

Rock failure processes can exhibit significant nonlinear behavior and characteristic failure mechanism transitions. During these processes, various failure mechanisms such as rock extension, shearing, and compaction yields can co-exist. Furthermore, rocks exhibit different failure modes under different confining pressures [1]. For example, under tensile stress or low-pressure conditions, rocks may exhibit tensile failure. As the pressure gradually increases, the failure mode transitions from tensile to shear failure. Similarly, under conditions of high temperatures and water exposure, rock materials such as sandstone and claystone exhibit significant changes in strength and failure mechanisms, as explored by Skrzypkowski et al. [2]. At higher stress levels in deeper regions, the failure mode shifts from conventional shear failure to pore collapse failure [3]. Thus, rocks transition between multiple failure modes, including tensile failure, shear failure, and compaction-shear yield.

Paul (1968) considered the influence of the intermediate principal stress on the Mohr-Coulomb model and proposed an expression involving three principal stresses [4,5]. Three parameter constants were determined for the stress states of uniaxial compression, uniform triaxial (isotropic) extension, and uniaxial extension. Meyer and Labuz further developed this into the PMC strength model [6]. They discussed the transformation characteristics of failure mechanisms in rock materials based on the PMC model and examined the shape of dodecagonal PMC failure surfaces. Two failure planes were fit to describe the nonlinear failure surface, which led to the development of the dodecahedral PMC model. This model allows for the observation of changes in the shape of the failure surface with increasing mean stress, characterizing the transition of rock material failure from tensile failure to shear failure as the stress conditions increase.

For the material parameters, Makhnenko et al. proposed a plane fitting method that minimizes the sum of the squared orthogonal distances between data points and the failure surface [7], whereas Zeng et al. suggested using the least squares method to fit parameters [8]. Asem et al. introduced a simplified PMC model that assumes the same friction angle under both tensile and compressive conditions and reduces the dodecagonal PMC model by two parameters; in other words, the resulting model has only four parameters [9].

Under tensile stress or low-pressure conditions, the rock failure mode is a tensile failure. Rocks undergo splitting failure under uniaxial stress or low confining pressure conditions with no tensile stress on the failure plane. Stacey (1981) proposed the extensional-strain criterion to explain the brittle failure mechanisms of deep quartzites in South Africa [10]. The results indicate that for materials exhibiting linear deformation behavior, the onset and depth of failure can be correlated with extensional strain.

This study characterizes rock failure under tensile and tensile-shear conditions using the extensional-strain criterion and describes compressive-shear failure based on the PMC model. A unified strength model under the influence of multiple failure mechanisms was then derived. By analyzing field and laboratory test data, a theoretical model suitable for multiple failure mechanisms in rocks was proposed, which revealed intrinsic low-strength rock failure mechanisms.

## 2. Related Work

### 2.1. PMC Model

In 1968, Paul considered the influence of the intermediate principal stress and proposed the PMC model [4,5]. In 2013, Meyer and Labuz developed this concept into the PMC strength model, and the expression for principal stresses is as follows [6]:

(1) $$A\sigma_{1}+B\sigma_{2}+C\sigma_{3}=1$$

where *A*, *B*, and *C* are material constants, and *σ*_1_, *σ*_2_, and *σ*_3_ represent the three principal stresses. When *B* = 0, Equation (1) becomes the MC (Mohr-Coulomb) strength criterion.

(2) $$A=\frac{1-\sin\varphi_{c}}{2\sin\varphi_{c}}\cdot \frac{1}{V_{0}}$$

(3) $$B=\frac{\sin\varphi_{c}-\sin\varphi_{e}}{2\sin\varphi_{c}\sin\varphi_{e}}\cdot \frac{1}{V_{0}}$$

(4) $$C=-\frac{1+\sin\varphi_{e}}{2\sin\varphi_{e}}\cdot \frac{1}{V_{0}}$$

where *φ_c_* and *φ_e_* are the internal friction angles during compression and extension, respectively, and *V*_0_ is the theoretical triaxial tensile strength.

By substituting the three parameters *A*, *B*, and *C* into Equation (1), they can be expressed in terms of the two internal friction angles and the vertex as follows:

(5) $$\left[\frac{1-\sin\varphi_{c}}{2\sin\varphi_{c}}\right]\cdot \frac{\sigma_{1}}{V_{0}}+\left[\frac{\sin\varphi_{c}-\sin\varphi_{e}}{2\sin\varphi_{c}\sin\varphi_{e}}\right]\cdot \frac{\sigma_{2}}{V_{0}}-\left[\frac{1+\sin\varphi_{e}}{2\sin\varphi_{e}}\right]\cdot \frac{\sigma_{3}}{V_{0}}=1$$

**Figure 1:**
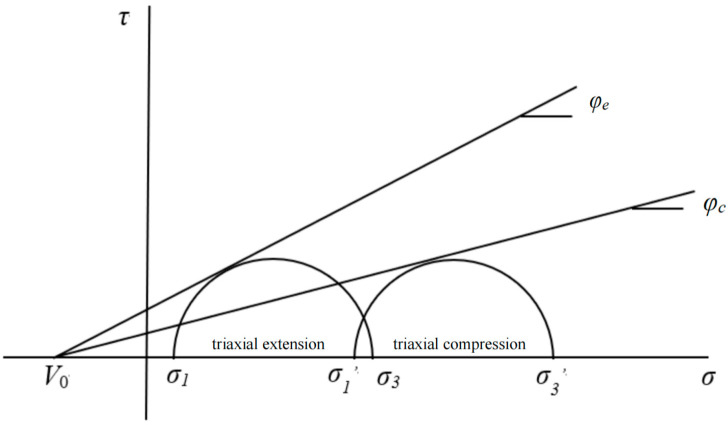
PMC model failure envelope. The internal friction angles during compression and extension are *φ_c_* and *φ_e_*, respectively. *V*_0_ is the theoretical triaxial tensile strength. The three principal stresses are *σ*_1_, *σ*_2_, and *σ*_3_.

The PMC failure model constructs a failure surface in the principal stress space with a common vertex *V*_0_. The PMC criterion accounts for the influence of the intermediate principal stress by using the different friction angles of rock materials under compression and tension. The multiaxial linear failure criterion includes three principal stresses. These three material parameters can be obtained using conventional triaxial tests on rocks. The PMC model failure envelope is shown in Figure 1.

**Figure 2:**
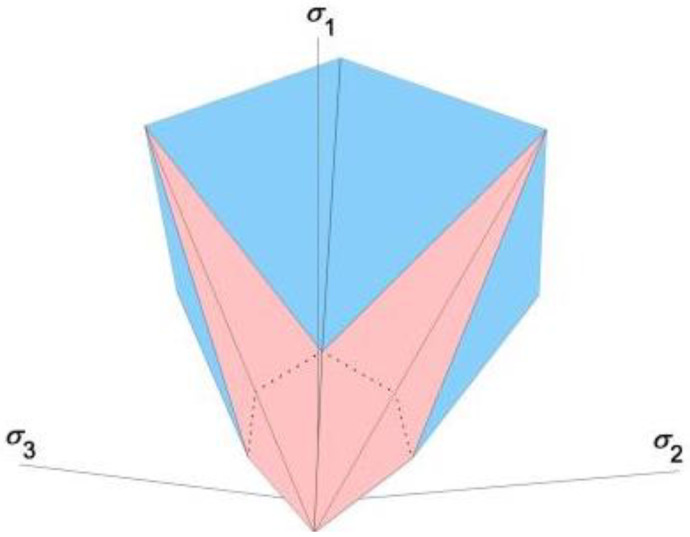
Dodecagonal PMC model. The dodecagonal failure surface, constructed in a piecewise linear manner by fitting two failure planes. The three principal stresses are *σ*_1_, *σ*_2_, and *σ*_3_.

To capture the transition characteristics of failure mechanisms in geotechnical materials, the Labuz team developed a dodecahedral PMC model based on the PMC framework [6]. The PMC failure criterion constructs a dodecagonal failure surface in a piecewise linear manner by fitting two planes (Figure 2), which can be approximately regarded as a nonlinear failure surface. This model characterizes the mechanism by which rock materials transition from tensile to shear failure under increasing stress conditions. When the constitutive model is applied to numerical simulations, the flow rule needs to be established [7].

### 2.2. Extensional-Strain Criterion and Brittle Failure

**Figure 3:**
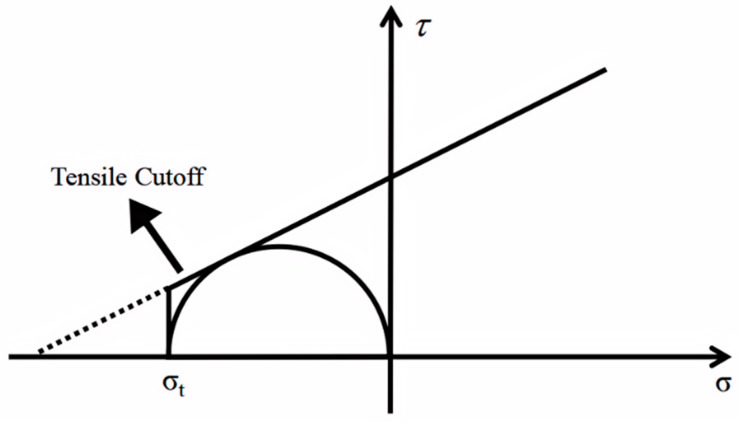
Schematic diagram of stretch truncation. The single inclined line of the MC criterion is modified to an inclined line and a vertical line tangent to the uniaxial tensile stress circle, forming a two-segment linear strength curve known as the tensile cutoff criterion. *σ_t_* represents the measured tensile strength.

Rocks exhibit different failure modes under different confining pressures. Under tensile stress or low-pressure conditions, rocks primarily undergo tensile failure, not shear failure. However, the MC and PMC models only include failure criteria under compressive and pure shear stress conditions and do not address failure criteria under tensile-shear conditions [11]. Therefore, using the MC criterion to calculate rock failure under tensile or low-stress levels often results in significant errors. To address this issue, Paul (1961) modified the single inclined line of the MC criterion to an inclined line and a vertical line tangent to the uniaxial tensile stress circle, forming a two-segment linear strength curve known as the tensile cutoff criterion. The modified failure criterion is shown in Figure 3 [12], where *σ_t_* represents the measured tensile strength. However, while the dodecagonal PMC model can characterize the mechanism of rock material transitioning from tensile failure to shear failure as stress conditions increase, it cannot represent the failure mechanism of rocks under tensile-shear conditions. Therefore, it is necessary to study the tensile-shear failure criterion of rocks and establish a connection with the PMC strength model.

Numerous scholars have investigated the strength criteria of rocks under tensile-shear conditions, and the resulting models can be roughly classified into hyperbolic models, parabolic models, and models based on the Hoek-Brown criterion [13,14,15,16,17,18,19]. However, relatively few studies have been conducted on this topic, and consequently, experimental data are lacking. Therefore, the tensile-shear constitutive models and failure criteria currently lack adequate support. Stacey (1981) predicted the brittle failure mechanisms of deep massive quartzites in South Africa using the extensional-strain criterion [10]. The results indicate that for materials exhibiting linear deformation behavior, failure may be related to extensional strain. The extensional strain of rocks can be calculated using the generalized Hooke’s law, as follows:

(6) $$\left\{\begin{array}{c} \varepsilon_{1}=\frac{1}{E}\;[\sigma_{1}-\mu (\sigma_{3}+\sigma_{2})]\\ \varepsilon_{2}=\frac{1}{E}\;[\sigma_{2}-\mu (\sigma_{1}+\sigma_{3})]\\ \varepsilon_{3}=\frac{1}{E}\;[\sigma_{3}-\mu (\sigma_{1}+\sigma_{2})] \end{array}\right.$$

where *μ* is the Poisson’s ratio; *E* is the elastic modulus; and *ε*_1_, *ε*_2_, and *ε*_3_ are the first, second, and third principal strains, respectively. According to the extensional-strain criterion, when the minimum principal stress-strain exceeds the critical strain, the rock undergoes tensile failure. When $\varepsilon_{3}<0$, the specimen experiences tensile strain, and when $\varepsilon_{3}>0$, the specimen experiences compressive strain.

## 3. Methodology

### 3.1. Proposal of the PMC Model Considering Extensional Strain

Based on the dodecagonal PMC model, a failure surface was added that considers the extensional-strain criterion. A three-segment strength formula that accounts for both tensile-shear and compressive-shear failures is proposed. This model can be expressed using the three principal stresses as follows:

(7) $$A\prime \sigma_{I}+B\prime \sigma_{\mathrm{II}}+C\prime \sigma_{\mathrm{III}}=1$$

where, *A*′, *B*′, and *C*′ are material constants; *σ*_1_, *σ*_2,_ and *σ*_3_ are the maximum, intermediate, and minimum principal stresses, respectively. For the failure surface, based on the generalized Hooke’s law, the failure conditions can be obtained as follows:

(8) $$\varepsilon_{1}\;or\;\varepsilon_{2}\;or\;\varepsilon_{3}=\varepsilon_{c}$$

where the critical strain $\varepsilon_{c}=-\sigma_{t}/E$ and is the tensile strength, *μ* is the Poisson’s ratio, and *E* is the elastic modulus. When $\sigma_{\mathrm{III}}-\mu (\sigma_{I}+\sigma_{\mathrm{II}})<0$, the rock is in the tensile failure stage or transitioning from tensile to shear failure. According to the extensional-strain criterion, when $\varepsilon_{1}=\varepsilon_{c}$, the rock undergoes tensile failure, and the following can be derived:

(9) $$-\sigma_{t}=\sigma_{\mathrm{III}}-\mu (\sigma_{I}+\sigma_{\mathrm{II}})$$

According to Equations (8) and (9):

(10) $$A\prime =B\prime =-\frac{\mu }{\sigma_{t}}$$

(11) $$C\prime =-\frac{1}{\sigma_{t}}$$

**Figure 4:**
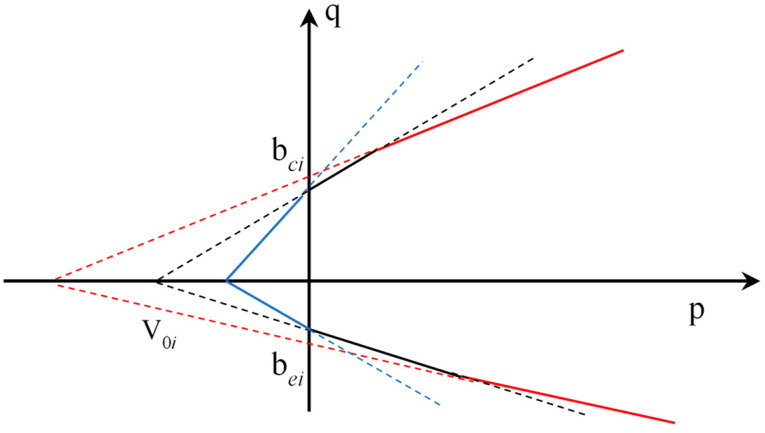
Extensional-strain criterion *p*-*q* plane schematic diagram. The linear forms represent the rock failure modes, from left to right: tensile failure, transition from tensile failure to shear failure, and conventional shear failure. The parameters in the figure, such as root and intercept, do not have specific meanings.

The three-segment linear form of the strength criterion can fundamentally satisfy the fitting of the transformation of rock failure mechanisms. Tensile or tensile-shear failure under tensile stress or low pressure is characterized by the extensional-strain criterion; as the pressure gradually increases, the rock failure mode transitions from tensile failure to shear failure; under higher stress levels, the rock failure mode transitions to conventional shear failure, as shown in Figure 4.

In the *p*-*q* plane, where the horizontal axis represents the mean stress *p* and the vertical axis represents the generalized shear stress *q*, the conventional PMC linear strength intersects the *q* axis at *b_ci_* and *b_e_*_i_ and intersects the *p* axis at *V*_0*i*_. In the *p*-*q* plane, Equation (7) can be written as follows:

(12) $$q=\frac{{b_{\theta }}_{i}}{{V_{0}}_{i}}p+b_{\theta i}=b_{\theta i}\left(\frac{p}{V_{0i}}+1\right)$$

where *b_θi_* is the intercept of the conventional PMC linear strength line in the *p*-*q* plane, and *V*_0*i*_ is its root.

By fitting the failure curve using a piecewise linear approach, a PMC strength formula that considers extensional strain can be obtained. In the *p*-*q* plane, the formula can be written as follows:

(13) $${q_{c}}^{\left(n\right)}=\min\left[b_{cn}\left(\frac{p}{V_{0i}}+1\right)\right]$$

(14) $${q_{e}}^{\left(n\right)}=\min\left[b_{en}\left(\frac{p}{V_{0i}}+1\right)\right]$$

where *n* represents the failure mechanism number and *q_c_*^(*n*)^ and *q_e_*^(*n*)^ are the critical shear stress values under different failure mechanisms. Similarly, *b_cn_* and *b_en_* represent the intercepts of the PMC linear strength lines on the *p*-*q* plane under different failure mechanisms, and *V*_0*i*_ represents their roots.

### 3.2. Unified Equation of the Multiple Failure Mechanism Model

Equation (14) is presented in the form of intercepts and other data, without specific expressive significance. For spatial loading, the influence of the Lode angle should be considered. Therefore, under the premise of spatial loading, deriving a model for multiple failure mechanisms is more effective. Based on the PMC criterion, a general model is proposed to obtain different solutions under various failure mechanisms.

In the PMC model, the strength from the meridional plane is extended to the stress space as follows:

(15) $$f=\sigma_{1}\left(\frac{1-sin\varphi_{c}}{2sin\varphi_{c}}\right)+\sigma_{2}\left(\frac{sin\varphi_{c}-sin\varphi_{e}}{2sin\varphi_{c}sin\varphi_{e}}\right)-\sigma_{3}\left(\frac{1+sin\varphi_{e}}{2sin\varphi_{e}}\right)-V_{0}=0$$

**Figure 5:**
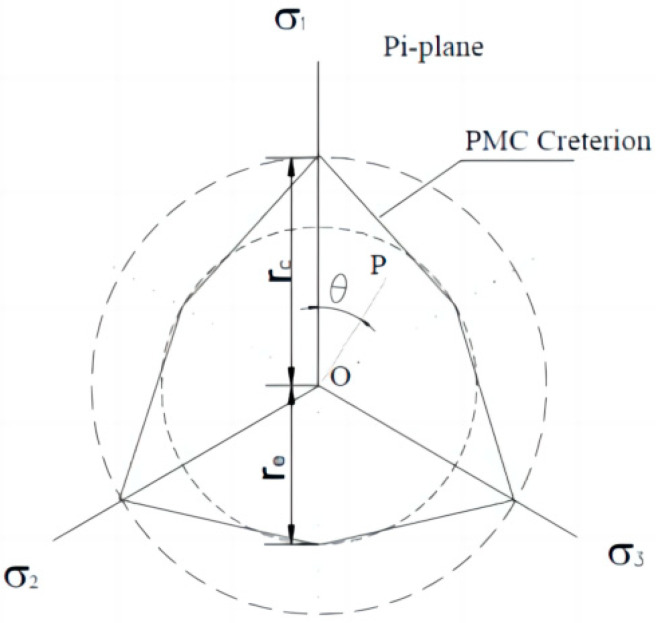
Relationship of the stress Lode angle and the π-plane. The maximum radius on the plane corresponds to the *r_c_* value under triaxial compression, and the minimum radius corresponds to the *r_e_* value under triaxial extension. *θ* represents the Lode angle.

It should be noted that this model assumes static loading conditions and does not account for dynamic loads. The loading rate in the experiment was referenced according to the method recommended by the International Society for Rock Mechanics [10]. Where *V*_0_ is the assumed strength under triaxial equal tensile conditions; *φ_c_* is the internal friction angle under triaxial compression; and *φ_e_* is the internal friction angle under triaxial extension. The triaxial compression and extension strength lines at the same *V*_0_ point can be considered a set, where *n* denotes the failure mechanism number. The definition of the stress Lode angle is shown in Figure 5. The Lode angle (0° ≤ *θ* ≤ 60°) is the rotation angle from the maximum principal stress axis (*θ* = 0°) to the minimum principal stress axis.

The maximum radius on the π-plane corresponds to the *r_c_* value under triaxial compression, and the minimum radius corresponds to the *r_e_* value under triaxial extension. Let the stress state at any point on this π-plane be (*p*, *q*, *θ*), where p is the mean stress, *q* is the generalized shear stress, and *θ* is the Lode angle. The relationship with the principal stresses is as follows:

(16) $$p=\frac{\sigma_{1}+\sigma_{2}+\sigma_{3}}{3}$$

(17) $$q=\sqrt{\frac{(\sigma_{1}-\sigma_{2}{)}^{2}+(\sigma_{2}-\sigma_{3}{)}^{2}+(\sigma_{3}-\sigma_{1}{)}^{2}}{2}}$$

As shown in Figure 5, with the centroid *O* of the π-plane as the center, the distance from any point on the plane to the center represents the magnitude of the deviatoric stress in the stress space. Taking the meridional plane as an example, its radius is determined as follows:

(18) $$r_{\theta }=(p+V_{0}){\tan\varphi }_{\theta }$$

where *r_θ_* and *φ_θ_* are the radius and the corresponding internal friction angle on the π-plane when the Lode angle is *θ*, and the connection line between the stress point and the far point is the slope line. When the stress state is triaxial compression, *φ_θ_* = *φ_c_*; when it is triaxial extension, *φ_θ_* = *φ_e_*.

On the π-plane, through the stress point (*p*, *q*, *θ*), perpendicular lines are drawn to the three coordinate axes, intersecting the axes at the points ($\sigma_{I}^{\prime }$, 0, 0), (0, $\sigma_{II}^{\prime }$, 0), and (0, 0, $\sigma_{III}^{\prime }$). Here, $\sigma_{I}^{\prime }$, $\sigma_{II}^{\prime }$, and $\sigma_{III}^{\prime }$ represent the principal stresses, but their magnitudes are not determined. In the quadrant shown in Figure 5, the order of the coordinates on the three axes is *σ*_1_ > *σ*_2_ > *σ*_3_. Roman numerals were used to indicate the order of the principal stress values. Thus:

(19) $$\sigma_{I}^{\prime }=r_{\theta }\cos\theta$$

(20) $$\sigma_{II}^{\prime }=r_{\theta }\cos(60^{{}^{\circ}}-\theta )$$

**Figure 6:**
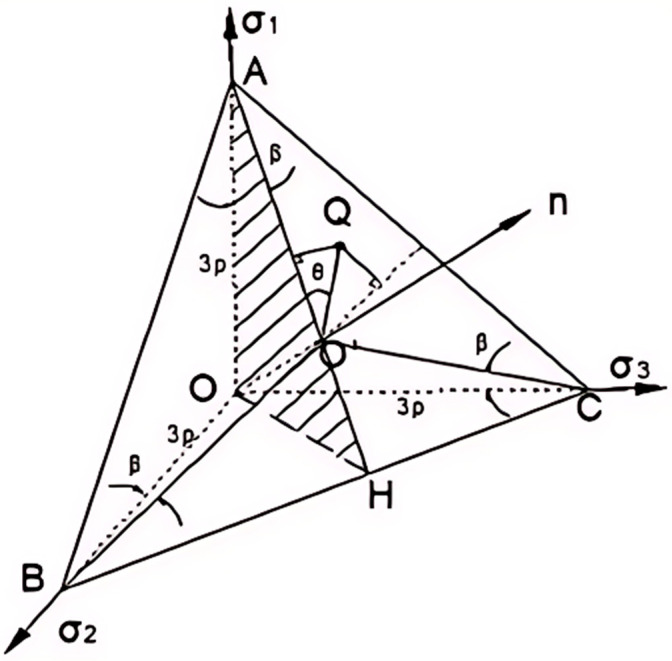
Relationship between the spatial and deviatoric stress radius on the π-plane. The length of the AH line segment is the height *h* of the equilateral triangle of the slope plane, and the intercepts of the slope plane along the three coordinate axes are all 3*p*. *θ* represents the Lode angle.

The relationship between the line segment on the π-plane and the principal stress space coordinates is shown in Figure 6. The length of the AH line segment is the height *h* of the equilateral triangle of the slope plane, and the intercepts of the slope plane along the three coordinate axes are all 3*p*. The geometric relationship yields:

(21) $$\frac{\sigma_{1}}{\sigma_{I}^{\prime }+\frac{h}{3}}=\frac{3p}{h}$$

Since the π-plane is an equilateral plane, $h=\frac{3\sqrt{6}}{2}p$. Substituting this into the equation yields $\sigma_{1}=\frac{\sqrt{6}}{3}\sigma_{I}^{\prime }+p$, and substituting into Equation (19) results in the following:

(22) $$\sigma_{1}=\frac{\sqrt{6}}{3}r_{\theta }\cos\theta +p$$

Similarly, the following can be obtained:

(23) $$\sigma_{2}=p-\frac{\sqrt{6}}{3}\sigma_{III}^{\prime }=p-\frac{\sqrt{6}}{3}r_{\theta }\cos(60^{{}^{\circ}}-\theta )$$

In the equation, *p* represents the mean stress. Since the principal stresses on the π-plane are equal, the stress value on the *σ*_3_ axis corresponds to the intermediate principal stress, which is as follows:

(24) $$\sigma_{3}=3p-\sigma_{1}-\sigma_{2}=p-\frac{\sqrt{6}}{3}r_{\theta }\left[\cos\theta -\cos\left(60^{{}^{\circ}}-\theta \right)\right]=p-\frac{\sqrt{6}}{3}r_{\theta }\cos\left(60^{{}^{\circ}}+\theta \right)$$

Substituting Equations (22)–(24) into Equation (15) and rearranging yields results in the following:

(25) $$\frac{\sqrt{6}}{3}r_{\theta }\;[\cos\theta \cdot \left(\frac{1-sin\varphi_{c}}{2sin\varphi_{c}}\right)-\cos\left(60^{{}^{\circ}}+\theta \right)\cdot \left(\frac{sin\varphi_{c}-sin\varphi_{e}}{2sin\varphi_{c}sin\varphi_{e}}\right)+\cos\left(60^{{}^{\circ}}-\theta \right)\cdot \left(\frac{1+sin\varphi_{e}}{2sin\varphi_{e}}\right)]-\left(p+V_{0}\right)=0$$

The relationship between the π-plane and the principal stress space shows that:

(26) $$r_{\theta }=(p+V_{0}){\tan\varphi }_{\theta }=\sqrt{\frac{2}{3}}q$$

In the equation, *q* is the generalized shear stress. Substituting Equation (26) into Equation (25) yields:

(27) $$\frac{2}{3}q\;[\cos\theta \cdot \left(\frac{1-sin\varphi_{c}}{2sin\varphi_{c}}\right)-\cos\left(60^{{}^{\circ}}+\theta \right)\cdot \left(\frac{sin\varphi_{c}-sin\varphi_{e}}{2sin\varphi_{c}sin\varphi_{e}}\right)+\cos\left(60^{{}^{\circ}}-\theta \right)\cdot \left(\frac{1+sin\varphi_{e}}{2sin\varphi_{e}}\right)]-\left(p+V_{0}\right)=0$$

Which can be rearranged as follows:

(28) $$q=\frac{6sin\varphi_{c}sin\varphi_{e}\left(p+V_{0}\right)}{2\sqrt{3}sin\varphi_{c}sin\theta -2sin\varphi_{c}sin\varphi_{e}\cos\left(60^{{}^{\circ}}+\theta \right)+2\sqrt{3}sin\varphi_{e}\sin\left(60^{{}^{\circ}}-\theta \right)}$$

When *θ* = 0°, the stress state corresponds to conventional triaxial compression. From Equation (28), the following expression is obtained:

(29) $$q=\frac{6{\sin\varphi }_{c}}{3-{\sin\varphi }_{c}}(p+V_{0})$$

When *θ* = 60°, the stress state corresponds to triaxial extension. From Equation (28), the following is obtained:

(30) $$q=\frac{6{\sin\varphi }_{e}}{3+{\sin\varphi }_{e}}(p+V_{0})$$

The calculation results are consistent with those obtained from the PMC strength Equation (15). Therefore, based on Equation (28), the general formula for the strength criterion in stress space under the coupled influence of multiple failure mechanisms can be derived as:

(31) $$q=min\left[\frac{6sin\varphi_{c}^{i}sin\varphi_{e}^{i}\left(p+V_{0}\right)}{2\sqrt{3}sin\varphi_{c}^{i}sin\theta -2sin\varphi_{c}^{i}sin\varphi_{e}^{i}\cos\left(60^{{}^{\circ}}+\theta \right)+2\sqrt{3}sin\varphi_{e}^{i}\sin\left(60^{{}^{\circ}}-\theta \right)}\right](i=1,\ldots ,n)$$

where *q* is the generalized shear stress; *p* is the mean stress; *θ* is the stress Lode angle (with the axis of the maximum principal stress as 0°); *φ_c_* is the internal friction angle under triaxial compression; *φ_e_* is the internal friction angle under triaxial extension; *V*_0_ is the assumed strength under triaxial equal tensile conditions; and *n* is the failure mechanism group.

**Figure 7:**
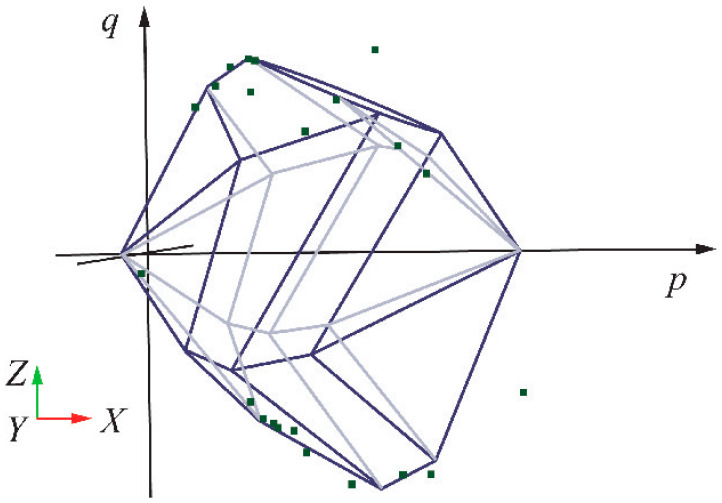
Model shape and data fitting in *p*-*q* space. The “cap”-shaped shear strength envelope during pore collapse in rocks.

When *n* = 1, the formula corresponds to the conventional PMC model; when *n* ≥ 2, the formula represents the multiple failure mechanism model. Specifically, when forming a yield model similar to a “cap”, the shear strength envelope can effectively explain the pore collapse phenomenon in porous rocks. Based on the data reported by Folta et al., the data fitting curve can be obtained as shown in Figure 7 [20]:

### 3.3. Parameter Fitting Method

Zeng et al. proposed a new fitting method based on the transformation of stress invariant relationships in the *p*-*q* plane [8,21]. They derived a parameter acquisition method for a multiple failure mechanism model based on this approach.

**Figure 8:**
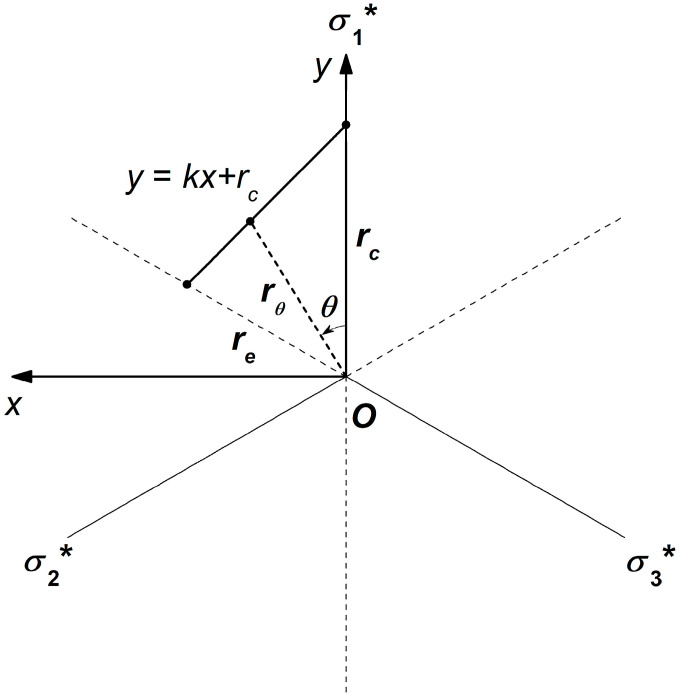
Schematic showing the PMC failure surface in the π plane with polar and orthogonal coordinate systems when *p* = 0. $y=kx+r_{c}$ represents the failure envelope line. *θ* represents the Lode angle, and *r_θ_* is the radius of the π-plane corresponding to the Lode angle *θ* and the slope line’s apical angle. *r_c_* is the intercept in the axisymmetric compression line, and *r_e_* represents the intercept in the multiaxial line. *σ*_1_*, *σ*_2_*, and *σ*_3_* represent the stress components in the transformed or cylindrical coordinate system within the π-plane. The * symbol indicates a specific stress state in the transformed cylindrical coordinate system within the π-plane.

In a π-plane, any given stress state can be represented in cylindrical coordinates (*r_θ_*, *θ*, *ρ*). A schematic of the PMC failure surface in the π-plane when *p* = 0 is shown in Figure 8.

The intercept *b_θ_* needs to be defined, and a relationship should be established between *b_θ_* and *r_θ_*. The failure envelope line $y=kx+r_{c}$ in the rectangular coordinate system can be expressed in terms of *r_θ_* and *θ* as follows:

(32) $$r_{\theta }\cos\theta =k\cdot r_{\theta }\sin\theta +r_{c}$$

where *θ* represents the Lode angle and *r_θ_* is the radius of the π-plane corresponding to the Lode angle *θ* and the slope line’s apical angle. *r_c_* is the intercept in the axisymmetric compression line. In the π-plane, *k* is the slope of the line, and *r_θ_* is as follows:

(33) $$r_{\theta }=\frac{r_{c}}{\cos\theta -k\sin\theta }$$

In any π-plane, the relationship between *r_θ_* and *q_θ_* can be obtained through the second stress invariant J_2_ as follows:

(34) $$r_{\theta }=\sqrt{\frac{2}{3}}q_{\theta }$$

where *q_θ_* is the shear stress in the direction of *r_θ_*. In the multiaxial line, *q_θ_* = *b_θ_*; in the axisymmetric compression line, *q_θ_* = *b_c_*, where *b_c_* is the shear stress intercept in the axisymmetric compression line. In the axisymmetric extension line, *q_θ_* = *b_θ_*. Therefore, *r_θ_* can be written as:

(35) $$\left\{\begin{array}{c} r_{\theta }=\sqrt{\frac{2}{3}}b_{\theta }\\ r_{c}=\sqrt{\frac{2}{3}}b_{c}\\ r_{e}=\sqrt{\frac{2}{3}}b_{e} \end{array}\right.$$

In the formula, *b_e_* represents the shear stress intercept in the axisymmetric extension line, and *r_e_* represents the intercept in the multiaxial line. Substituting Equation (35) into Equation (33) yields *b_e_* as follows:

(36) $$b_{\theta }=\frac{b_{c}}{\cos\theta -k\sin\theta }$$

In the *p*-*q* plane, Equation (36) represents the functional relationship of the intercept *b_θ_* and the unknown variable *k* needs to be determined. In the π-plane, where the failure line passes through the axisymmetric extension point (*r_θ_* = *r_e_*, *θ* = 60°), Equation (32) can be written as:

(37) $$r_{e}=\sqrt{3}\cdot k\cdot r_{e}+2r_{c}$$

Considering Equation (35), the ratio $\alpha =\frac{r_{c}}{r_{e}}$ can be written as:

(38) $$\alpha =\frac{r_{c}}{r_{e}}=\frac{\sqrt{\frac{2}{3}}\cdot b_{c}}{\sqrt{\frac{2}{3}}\cdot b_{e}}=\frac{b_{c}}{b_{e}}$$

Substituting Equation (38) into Equation (37) yields:

(39) $$k=\frac{1-2\alpha }{\sqrt{3}}$$

Knowing the expressions for *b_θ_* and *k*, the general Equation (39) for the PMC criterion in the *p*-*q* plane can be written as:

(40) $$\frac{b_{c}}{V_{0}}p+kq\sin\theta +b_{c}=q\cos\theta$$

According to Equation (40), a linear equation system can be constructed using the data from axial compression, extension, and multiaxial tests, with *θ* under various conditions. In some studies, *θ* is equivalent to the Lode angle, but differences exist. The original definition of the Lode angle ranges from −30° to 30°, but in the coordinate system, *θ* ranges from 0° to 360°. However, *θ* ranging from 0° to 60° is sufficient for isotropic expressions. Whereas *θ* = 0° corresponds to axisymmetric compression and *θ* = 60°corresponds to axisymmetric extension. The *θ* value in multiaxial stress states can be calculated from the principal stresses as follows:

(41) $$\tan\theta =\frac{\sqrt{3}\left(\sigma_{\mathrm{II}}-\sigma_{\mathrm{III}}\right)}{2\sigma_{I}-\sigma_{\mathrm{II}}-\sigma_{\mathrm{III}}}$$

where $\sigma_{I}$,${\;\sigma }_{\mathrm{II}}$, and $\sigma_{\mathrm{III}}$ represent the maximum, intermediate, and minimum principal stresses, respectively. Strength data under axisymmetric and multiaxial conditions are expressed as $\sigma_{I},{\;\sigma }_{\mathrm{II}},and\sigma_{\mathrm{III}},$ and by substituting these into Equation (40), the *p*, *q*, and *θ* for each stress state can be determined. Using these three parameters, a system of linear equations *A*·*x* = *B* is generated:

(42) $$\left(\begin{array}{ccc} p_{1} & q_{1}{\sin\theta }_{1} & 1\\ p_{2} & q_{2}{\sin\theta }_{2} & 1\\ \vdots & \vdots & \vdots \\ p_{n} & q_{n}{\sin\theta }_{n} & 1 \end{array}\right)\left(\begin{array}{c} \frac{b_{c}}{V_{0}}\\ k\\ b_{c} \end{array}\right)=\left(\begin{array}{c} q_{1}{\cos\theta }_{1}\\ q_{2}{\cos\theta }_{2}\\ \vdots \\ q_{n}{\cos\theta }_{n} \end{array}\right)$$

where *A* is the rectangular data matrix, *x* is the parameter vector, and *B* is the data vector.

Using the least squares method to solve Equation (42), *b_c_*/*V*_0_, *k*, and *b* are obtained. According to the expressions for the axisymmetric compression and extension lines in the *p*-*q* plane, *φ_c_* and *φ_e_* are calculated as follows:

(43) $$\left\{\begin{array}{c} \sin\varphi_{c}=\frac{3b_{c}}{6V_{0}+b_{c}}\\ \sin\varphi_{e}=\frac{3b_{e}}{6V_{0}-b_{e}} \end{array}\right.$$

Thus, the material parameters *φ_c_*, *φ_e_*, and *V*_0_ of the general formula for the strength criterion in stress space under the coupled influence of multiple failure mechanisms (Equation (31)) can be solved.

## 4. Experiments

### 4.1. Experimental Conditions

Experiments were conducted on Sichuan yellow sandstone and deep granite from the Jinchuan No. 2 mining area. High-pressure triaxial compression tests were performed using the TAW-2000 microcomputer-controlled electro-hydraulic servo rock triaxial testing machine and the portable self-sealing rock triaxial test high-pressure chamber pressurization system.

Standard rock mechanics specimens with dimensions of *φ*30 mm × 60 mm were prepared. The preparation of rock samples follows the ISRM-suggested method for the complete stress-strain curve under rigid experimental conditions [22]. For each set of confining and axial pressures, only one set of experimental results is selected. If there is a significant deviation in the results, the experiment will be repeated.

This conventional triaxial test relies on two independent loading systems and therefore cannot use the system settings to load axial and confining pressures simultaneously at the same rate. To ensure quasi-static conditions and reduce experimental error, an independent stepwise loading method for axial and confining pressures was adopted. The stepwise loading procedure is shown in Table 1. During the experiment, axial loading was initially controlled by stress, with a loading rate of 100 N/s. After reaching the hydrostatic pressure state, the radial pressure was maintained at a constant level throughout the experiment. Axial loading was then continued at the same rate until approximately 50% of the peak strength was reached, at which point it was switched to deformation control with a loading rate of 0.03 mm/min. During confining pressure loading, coarse control was first applied, with a loading rate of 1 MPa/s. When approaching 5 MPa of the hydrostatic pressure state, fine control was applied using a micro-adjustment handwheel with a loading rate of 0.2 MPa/s.

### 4.2. Experimental Results

**Figure 9:**
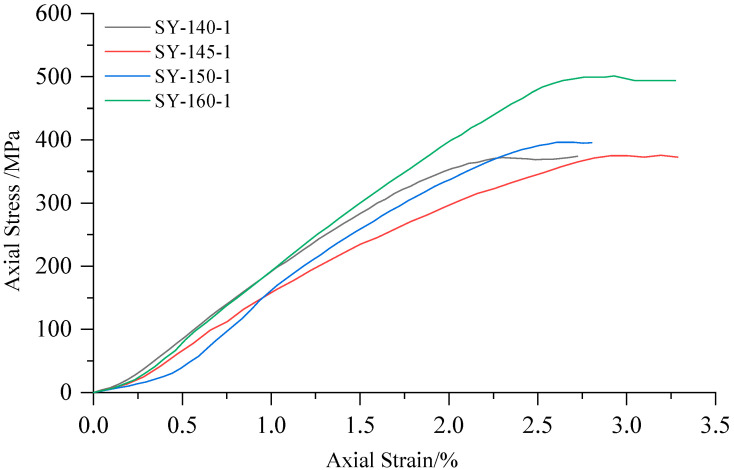
Stress-strain curves of Sichuan yellow sandstone in conventional triaxial compression tests.

**Figure 10:**
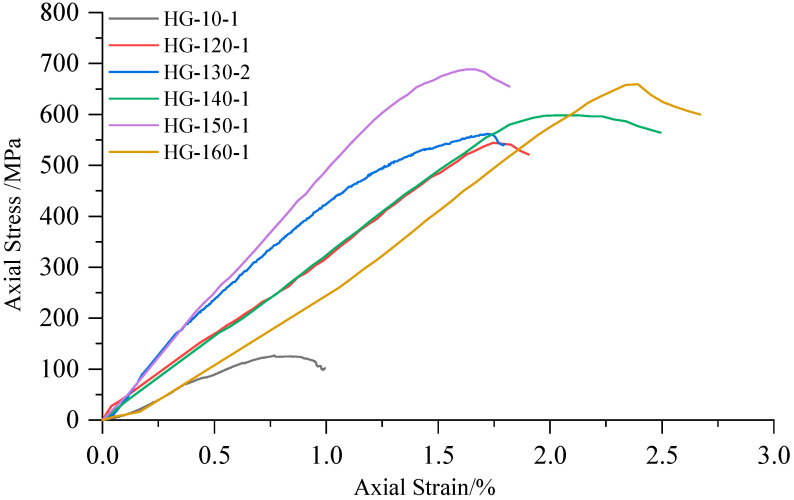
Stress-strain curves of Jinchuan granite in conventional triaxial compression tests.

**Figure 11:**
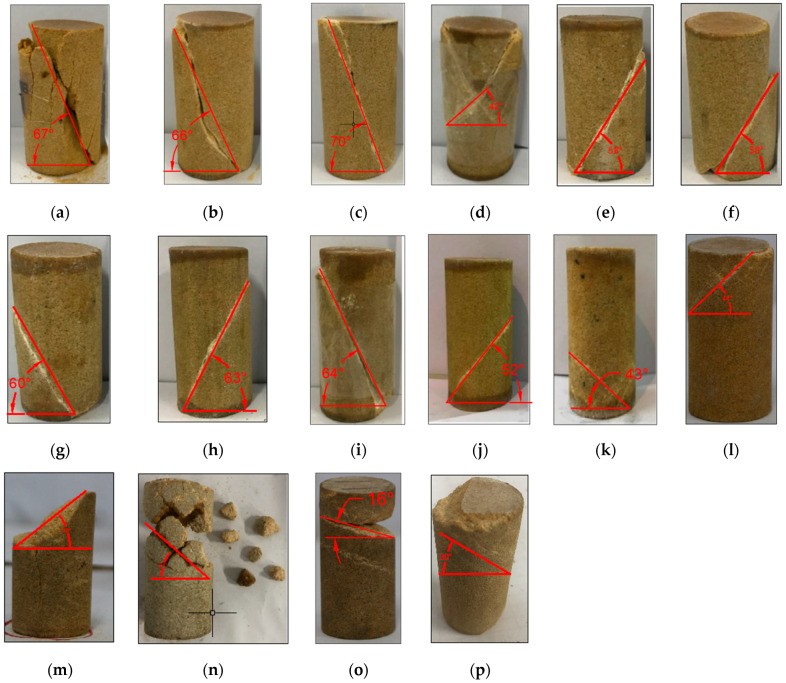
Failure modes of Sichuan yellow sandstone: (**a**) SY-0-1; (**b**) SY-10-1; (**c**) SY-20-1; (**d**) SY-30-1; (**e**) SY-40-1; (**f**) SY-50-1; (**g**) SY-60-2; (**h**) SY-65-1; (**i**) SY-70-1; (**j**) SY-90-1; (**k**) SY-110-2; (**l**) SY-140-2; (**m**) SY-145-1; (**n**) SY-150-1; (**o**) SY-160-1; (**p**) SY-170-1. (**a**–**p**) show the failure plane angles with gradually increasing confining pressure.

**Figure 12:**
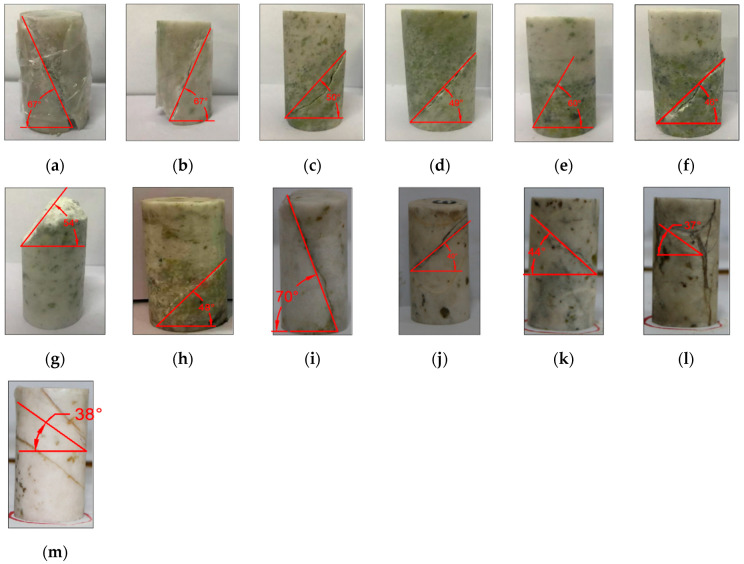
Failure modes of Jinchuan granite: (**a**) HG-0-1; (**b**) HG-10-1; (**c**) HG-20-1; (**d**) HG-30-1; (**e**) HG-40-2; (**f**) HG-50-1; (**g**) HG-60-1; (**h**) HG-100-1; (**i**) HG-120-1; (**j**) HG-130-2; (**k**) HG-140-1; (**l**) HG-150-1; (**m**) HG-160-1. (**a**–**m**) show the failure plane angles with gradually increasing confining pressure.

High-confining-pressure conventional triaxial tests were conducted on Sichuan yellow sandstone and Jinchuan granite using the above stepwise loading scheme. For the Sichuan sandstone, 16 groups of different confining pressures ranging from 0 to 170 MPa were set, whereas for the Jinchuan granite, 13 groups of different confining pressures ranging from 0 to 160 MPa were set. The stress-strain curves for each rock sample during the tests are shown in Figure 9 and Figure 10. The data for principal stress, mean stress, and deviatoric stress at failure are presented in Table 2. The failure modes of the samples are shown in Figure 11 and Figure 12. Both the Sichuan yellow sandstone and Jinchuan granite exhibited similar failure mode trends with increasing confining pressure: under low confining pressure, the failure is primarily shear failure, with the angle of the failure plane decreasing as the confining pressure increases; whereas under higher confining pressure, the angle continues to decrease, gradually exhibiting a trend towards compressive-shear failure.

### 4.3. Applicability of the PMC Model for Strength with Multiple Failure Mechanisms

**Figure 13:**
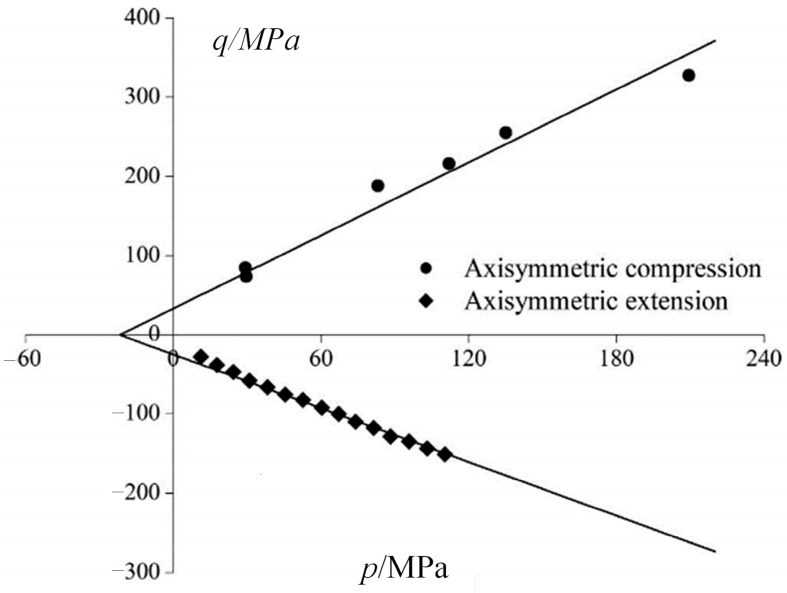
Experimental results obtained for the Berea sandstone. The negative axis of *q* represents triaxial extension tests, and the positive axis of *q* represents triaxial compression tests.

When *n* = 1, analysis is conducted using Berea sandstone experimental data (Bobich) (Table 3) [23]. The fitted failure surface in the *p*-*q* plane with the experimental data (conventional triaxial compression tests and conventional triaxial extension tests) is shown in Figure 13. The negative axis of *q* represents triaxial extension tests, and the positive axis of *q* represents triaxial compression tests. The PMC criterion fitting results closely match the experimental results, and the friction angle in conventional triaxial compression tests is smaller than in conventional triaxial extension tests. The difference between *φ_c_* and *φ_e_* illustrates the influence of the intermediate principal stress. Similarly, the study by Guo et al. explored the failure process of heterogeneous brittle rocks under uniaxial compression, highlighting the significant role of material heterogeneity in stress redistribution and crack formation [24]. Additionally, Wen et al. conducted qualitative and quantitative investigations on the effect of critical fissures on the failure process of rock specimens under plane strain compression, further emphasizing the importance of crack evolution in determining rock strength [25].

**Figure 14:**
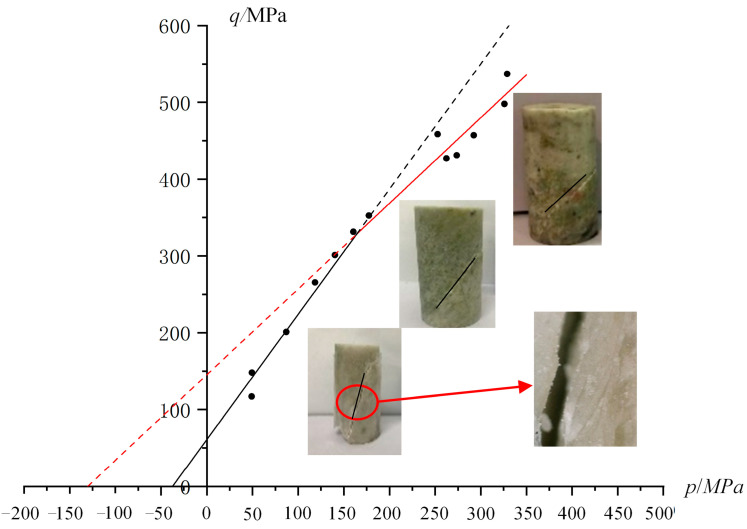
Six-parameter PMC model for granite on the *p*-*q* plane. The six parameters represent the failure mechanism number *n* for *n* = 1 and *n* = 2 as *φ_c_*, *φ_e_*, and *V*_0_. The piecewise linear relationship shown in the figure represents the results of the PMC six-parameter fitting. The diagram in the figure illustrates the cracking of Jinchuan granite, with the cracking plane angles gradually increasing from lower left to upper right as the confining pressure increases.

When *n* = 2, the experimental data for Jinchuan granite samples are analyzed. To ensure *V*_0_^(1)^ ≥ *V*_0_^(2)^, the region is divided into two segments: *p* ≥ 200 MPa and *p* ≤ 200 MPa. The six-parameter PMC model fitting results are shown in Table 4. The six-parameter PMC model for granite in the *p*-*q* plane is shown in Figure 14. The granite strength exhibits distinct piecewise linear characteristics, which can be fitted using the six-parameter PMC model. However, due to the lack of triaxial extension test data, this model is only applicable to the transitional stage of tensile-shear failure in rocks and cannot characterize the tensile failure mode of granite.

**Figure 15:**
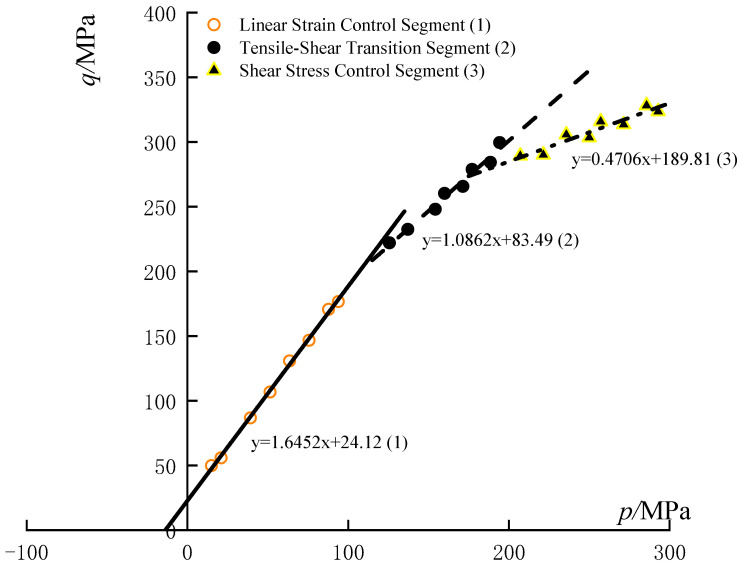
Fitting the failure strength of yellow sandstone with multiple mechanisms. The diagram shows the comparison between the PMC-fitted failure surface and the experimental data curves in the *p*-*q* plane.

When *n* > 2, the experimental data for Sichuan yellow sandstone samples are analyzed, and the fitting results are shown in Table 5. The fitted PMC failure surface with the experimental data in the *p*-*q* plane is shown in Figure 15. The strength of the yellow sandstone exhibits distinct piecewise strength characteristics, which conform to strength patterns under the influence of multiple failure mechanisms (Figure 15).

## 5. Conclusions

The rock multiple failure mechanism theory model proposed in this paper addresses the limitations of considering only a single failure mechanism. The model introduces a unified expression through the PMC piecewise linear model to describe multiple failure modes, including tensile, splitting, and shear failures. It integrates established criteria such as the Mohr-Coulomb strength theory and the 12-sided PMC model and extends to include the asymmetric PMC-BPMC model providing a comprehensive tool for analyzing complex failure processes in rock materials. The model’s applicability is supported by experimental validation using data from Jinchuan granite and Sichuan yellow sandstone.

(1) In light of the limitations of traditional rock strength theories, which consider only the impact of a single failure mechanism, and recognizing that tensile and splitting failures of rocks under low-stress conditions conform to the maximum elongation strain theory while shear-type failures occur at high-pressure levels, a multiple failure mechanism theory model for rocks has been proposed based on the PMC piecewise linear model. This model establishes a unified expression for multiple failure modes in the form of inequalities. Experimental verification has demonstrated that this criterion can effectively achieve nonlinear fitting for granite, sandstone, and other rock types.
(2) The rock multiple failure mechanism theory model’s failure criterion can encompass the MC strength theory, the PMC strength model, and the 12-sided PMC model. It can also be extended to derive the asymmetric PMC-BPMC model, which considers isotropic yield. By integrating the principles of plastic flow rules and hardening laws, the model can be expanded to analyze and explain the mechanisms of pore collapse phenomena in rocks under high pressure, corresponding to the specialized “cap” yield model mentioned in the text.
(3) Using experimental data from Jinchuan granite and Sichuan yellow sandstone, the rock multiple failure mechanism theory model was fitted for *n* = 1 (considering only shear failure), *n* = 2 (considering the transition from shear failure to tensile failure), and *n* > 2 (coexistence of tensile and shear failure). The results indicate that the rock strength exhibits distinct segmented strength characteristics, consistent with the strength patterns under the influence of multiple failure mechanisms. This validates the applicability of the multiple failure mechanism theory model to rock materials. However, since this study only considers generalized shear stress, mean stress, and stress Lode angle parameters in the rock failure process, other minor stress influences and environmental factors were not discussed, presenting certain limitations.

## Figures and Tables

**Table 1:** Comparison table of the axial load and axial stress under step loading.

*σ*/MPa	*P*/kN	*σ*/MPa	*P*/kN	*σ*/MPa	*P*/kN
5	3.5	65	45.5	125	87.5
10	7	70	49	130	91
15	10.5	75	52.5	135	94.5
20	14	80	56	140	98
25	17.5	85	59.5	145	101.5
30	21	90	63	150	105
35	24.5	95	66.5	155	108.5
40	28	100	70	160	112
45	31.5	105	73.5	165	115.5
50	35	110	77	170	119
55	38.5	115	80.5	175	122.5
60	42	120	84	180	126

**Table 2:** Summary of the conventional triaxial compression analysis of Sichuan yellow sandstone and Jinchuan granite.

Number	Wave Velocity/km/s	*σ*_2_/MPa	*σ*_3_/MPa	*σ*_1_/MPa	*p*/MPa	*q*/MPa	$\theta_{l}$/°
SY-0-1	1.43	0	0	49	16	49	−30
SY-10-1	1.58	10	10	107	42	97	−30
SY-20-1	1.69	20	20	173	71	153	−30
SY-30-1	1.42	30	30	222	94	192	−30
SY-40-1	1.54	40	40	219	100	179	−30
SY-50-1	1.43	50	50	267	122	217	−30
SY-60-2	1.5	60	60	274	131	214	−30
SY-65-1	1.52	65	65	322	151	257	−30
SY-70-1	1.46	70	70	361	167	291	−30
SY-90-1	1.46	90	90	398	193	308	−30
SY-110-2	1.72	110	110	377	199	267	−30
SY-140-2	1.43	140	140	430	237	290	−30
SY-145-1	1.61	145	145	477	256	332	−30
SY-150-1	1.46	150	150	460	253	310	−30
SY-160-2	1.62	160	160	502	274	342	−30
SY-170-1	1.45	170	170	490	277	320	−30
HG-0-1	8.78	0	0	148	49	148	−30
HG-10-1	8.33	10	10	127	49	117	−30
HG-20-1	8.56	20	20	221	87	201	−30
HG-30-1	8.33	30	30	296	119	266	−30
HG-40-2	8.45	40	40	341	140	301	−30
HG-50-1	8.78	50	50	381	160	331	−30
HG-60-1	8.36	60	60	413	178	353	−30
HG-100-1	7.89	100	100	559	253	459	−30
HG-120-1	8.33	120	120	547	262	427	−30
HG-130-2	8.38	130	130	561	274	431	−30
HG-140-1	8.82	140	140	597	292	457	−30
HG-150-1	7.14	150	150	687	329	537	−30
HG-160-1	8.33	160	160	658	326	498	−30

**Table 3:** PMC model fitting results.

Material	*φ_c_*/°	*φ_e_*/°	*V*_0_/kPa
Berea Sandstone	37.7	44.2	21.7

**Table 4:** Parameters used in the Jinchuan granite PMC model.

Failure Mechanism Number *n*	*φ_c_*/°	*φ_e_*/°	*V*_0_/kPa
1	42.853	42.853	27.712
2	28.020	28.020	130.953

Note: Due to the lack of triaxial extension and true triaxial tests, only triaxial compression data are used for fitting; therefore, *φ_c_* = *φ_e_*.

**Table 5:** Strength parameters of sandstone under the influence of multiple failure mechanisms.

Failure Mechanism Number *n*	*V*_0_/MPa	*φ_c_*/°	*φ_e_*/°
1 (low-pressure failure segment)	14.66	40	40
2 (transition segment)	76.87	27	27
3 (high-pressure failure segment)	403.31	13	13

Note: Due to the lack of triaxial extension and true triaxial tests, only triaxial compression data are used for fitting; therefore, *φ_c_* = *φ_e_*.

## Data Availability

The authors have not obtained permission to publish the data. Therefore, the data can be obtained from the corresponding author upon reasonable request.
